# Successful Hemostasis With Direct Clipping Using a Tapered‐Tip Hood and Slim Endoscope for Colonic Diverticular Bleeding Refractory to Endoscopic Band Ligation: A Case Report

**DOI:** 10.1002/deo2.70439

**Published:** 2026-09-23

**Authors:** Hirotake Kusumoto, Toru Kuwano, Yu Horikawa, Yukihiro Okada, Mayumi Iguchi, Yukiko Baba, Hideyuki Kishita, Koichiro Tsukasa, Shunji Shimaoka

**Affiliations:** ^1^ Department of Gastroenterology Nanpuh Hospital Kagoshima Japan

**Keywords:** colonic diverticular bleeding, direct clipping, endoscopic band ligation, endoscopic hemostasis, tapered‐tip hood

## Abstract

Endoscopic band ligation (EBL) is performed for hemostasis in patients with colonic diverticular bleeding (CDB) due to its low rebleeding rate. However, suction and ligation are not successful in some cases. We describe the case of a 93‐year‐old woman with CDB in whom EBL failed. Because of tissue rigidity, the sigmoid diverticulum could not be adequately suctioned into the ligation device, which resulted in band detachment and subsequent rebleeding. A slim endoscope fitted with a calibrated, small‐caliber‐tip, transparent hood enabled visualization of the bleeding point within the diverticulum. Complete hemostasis was then achieved by direct clipping using a reopenable clip. This case suggests that direct clipping using a tapered‐tip hood with a slim endoscope can be a useful rescue technique after unsuccessful EBL.

## 1 Introduction

Colonic diverticular bleeding (CDB) is a common cause of lower gastrointestinal bleeding. However, prompt identification of the bleeding point and reliable hemostasis can be challenging. The use of a distal attachment hood or a water‐jet scope is useful for identifying the bleeding point [1, 2, 3], and various methods are employed to achieve hemostasis, such as clipping and endoscopic band ligation (EBL). EBL involves suctioning the bleeding diverticulum into a dedicated hood attached to the endoscope tip, followed by deployment of an O‐band to ligate the diverticulum. If the diverticulum is adequately suctioned, ligation can be performed without complex techniques. However, in some cases, suction and ligation are not achieved owing to factors like tissue rigidity [4]. Here, we report a case of CDB in which EBL failed to achieve hemostasis. However, we successfully visualized the bleeding point using a tapered‐tip hood and slim endoscope, achieving hemostasis by direct clipping.

## 2 Case Report

**FIGURE 1:**
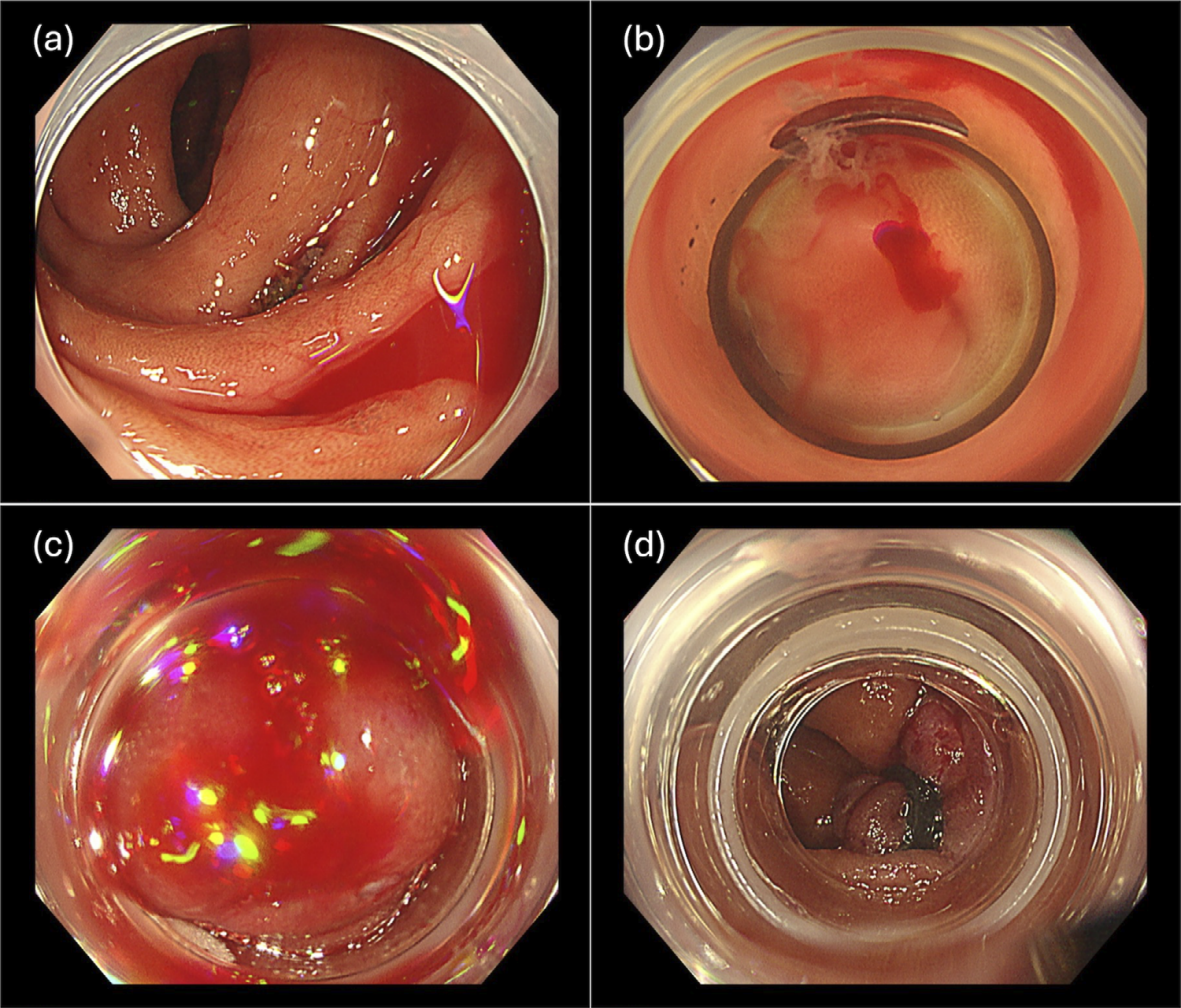
Colonic diverticular bleeding and failed EBL. (a) Active bleeding was observed in the sigmoid colon. (b) Active bleeding from the diverticulum in the sigmoid colon was visualized through the EBL device. (c) A direct view of the area, centered on the diverticulum, was obtained, and firm suction was performed. (d) The O‐band engaged the diverticulum only superficially and detached easily upon contact. CDB, colonic diverticular bleeding; EBL, endoscopic band ligation.

**FIGURE 2:**
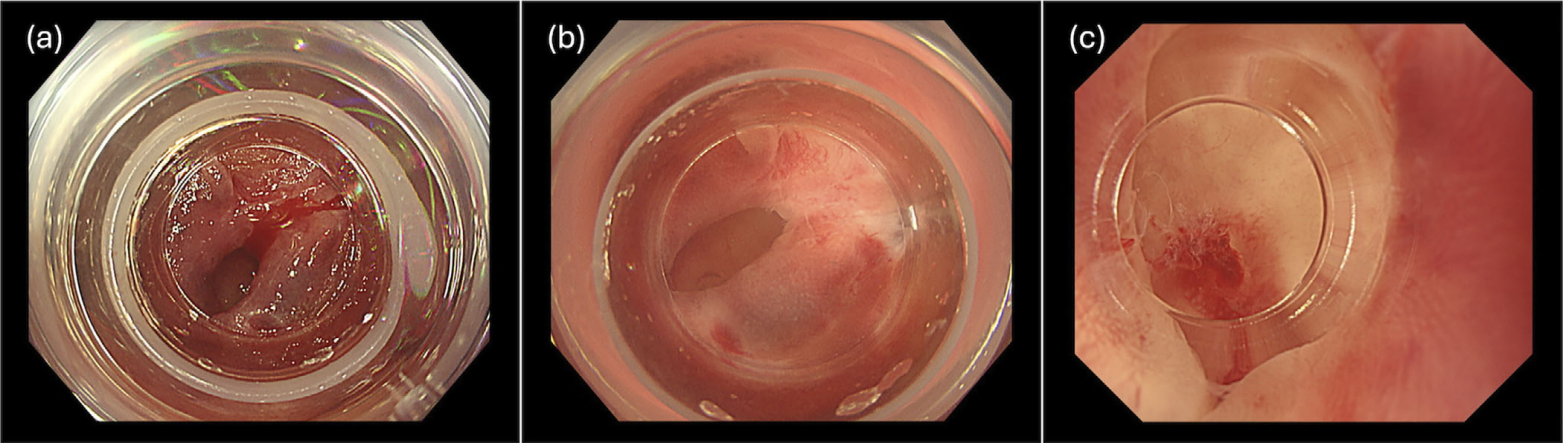
Visualization of the bleeding point. (a) The bleeding point was not visible with the long hood under carbon dioxide insufflation. (b) No bleeding point was visible, even after changing water immersion conditions. Scar‐like whitish changes were observed at the margin of the diverticulum. (c) The slim scope with a CAST hood allowed visualization of the exposed vessel and bleeding point.

**FIGURE 3:**
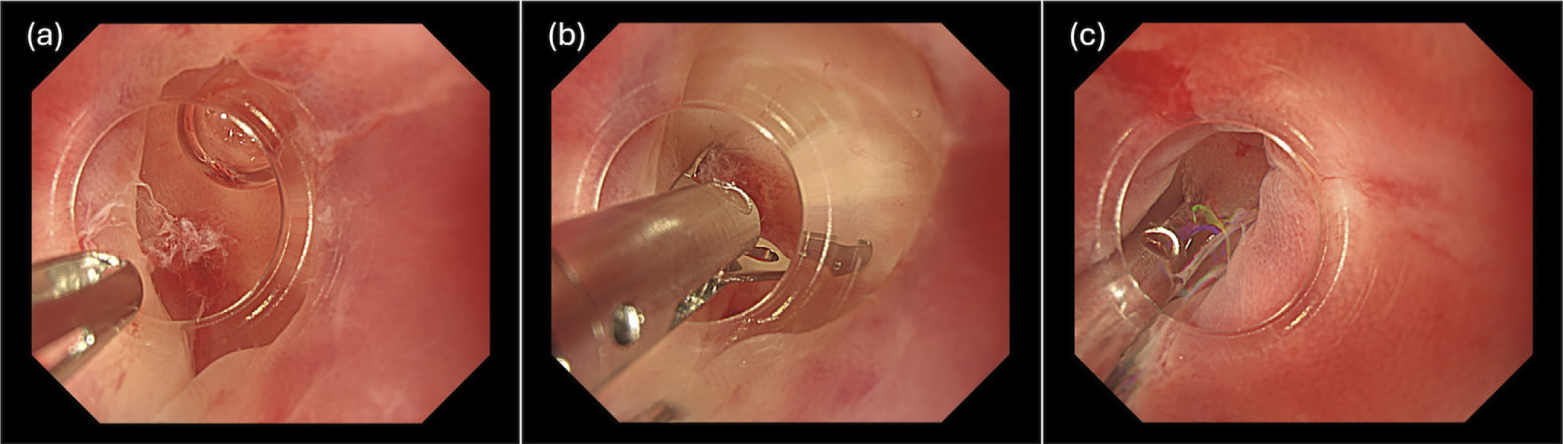
Direct clipping with the reopenable clip. (a) The reopenable clip emerged from the forceps channel while the bleeding point remained in view. (b) The clip was opened without changing the scope's position. (c) Direct clipping of the exposed vessel was completed.

A 93‐year‐old woman with massive hematochezia was admitted to our hospital. She had a history of multiple episodes of CDB and myocardial infarction treated with percutaneous coronary intervention and was taking low‐dose aspirin. She had no abdominal pain, and her vital signs were stable, with the shock index score remaining below 1. After admission, the patient was initially managed with bed rest and intravenous nutrition. However, bloody stools persisted, and laboratory data showed a decrease in the hemoglobin level from 12.5 to 10.8 g/dL. Although blood transfusion was not required, colonoscopy was performed after standard bowel preparation. First, we carefully searched for the bleeding source using a PCF‐H290ZI (Olympus, Tokyo, Japan) fitted with a conventional hood and identified an actively bleeding diverticulum in the sigmoid colon (Figure 1a). Next, we placed a marking clip near the diverticulum and performed EBL. Although several trial suction attempts were made while viewing the diverticulum as directly from the front as possible (Figure 1b) and applying sufficient suction each time, we were unable to draw an adequate amount of tissue into the hood (Figure 1c). Although the O‐band was deployed at the point of maximal suction, it was positioned only superficially over the diverticulum (Figure 1d). The band came off with a slight touch, causing rebleeding. We then tried to look for and capture the bleeding point in the diverticulum using a long hood after firing the O‐band. However, adequate visualization of the diverticular interior was impossible because the hood's outer diameter was larger than that of the diverticular orifice (Figure 2a,b). Therefore, we replaced the colonoscope with a slim upper gastrointestinal endoscope (GIF‐XZ1200; Olympus, Tokyo, Japan) fitted with a calibrated, small‐caliber‐tip, transparent (CAST) hood (TOP, Tokyo, Japan). Using the clip placed as a landmark during EBL, we advanced the endoscope to the culprit diverticulum. The tapered tip of the CAST hood was then inserted into the diverticulum, and we maintained the diverticular distension using the forward water‐jet function to prevent collapse and secure an adequate view. The CAST hood enabled us to look inside the diverticulum and clearly visualize the bleeding point under mild magnification (Figure 2c). Hemostasis was successfully achieved using a reopenable clip (SureClip 8 mm; MICRO‐TECH (Nanjing) Co., Ltd., Nanjing, China) (Figure 3). The total procedure time was 60 min; the cecal intubation time was 8 min, and the culprit diverticulum was identified 13 min after insertion. Only 2 min elapsed from reaching the culprit diverticulum to visualizing the bleeding point within it and achieving complete hemostasis. The patient was discharged on day 5, and no rebleeding occurred within 90 days.

## 3 Discussion

Various endoscopic hemostatic techniques have been reported for CDB, including clipping, ligation, coagulation, and epinephrine injection. Clipping methods include direct clipping, which involves directly grasping the bleeding point, and indirect clipping, which involves closing the diverticular orifice. Direct clipping is associated with a lower rebleeding rate than indirect clipping [2, 5]. However, in a study comparing EBL with the clipping method, EBL demonstrated superior outcomes for early rebleeding within 30 days, late rebleeding within 1 year, conversion to arterial embolization, and length of hospital stay [2, 6]. Based on these findings, our institutional strategy for CDB is to attempt hemostasis with EBL or direct clipping whenever possible. EBL is a highly versatile technique, although it can be challenging in some cases. Several factors have been reported to contribute to EBL failure, including a hard or fibrotic diverticulum, scarring from previous hemostatic treatment, a small diverticular orifice, and lesions that cannot be visualized en face using a tangential approach [4]. In the present case, underwater observation using a long hood revealed scar‐like whitish changes at the margin of the diverticulum, suggesting that tissue rigidity resulted in inadequate suction.

There is limited evidence regarding second‐line treatment after an unsuccessful EBL. Yamazaki et al. reported a case in which EBL failed because the tissue was too rigid to allow sufficient suction, whereas hemostasis was subsequently achieved using an over‐the‐scope clip (OTSC) [7]. Although OTSC may be effective in cases with rigid diverticular tissue, its relatively high cost may limit its widespread use.

Methods for directly visualizing an exposed vessel within a diverticulum using an ultrathin endoscope [8] or a CAST hood [9, 10] have been reported. However, the hemostatic devices available for use with an ultrathin endoscope are limited. Among the two reported cases in which the bleeding point was identified using a CAST hood, hemostasis was achieved by EBL in one case [9], whereas in the other, coagulation hemostasis was performed with the CAST hood remaining attached, followed by closure of the diverticulum using the reopenable‐clip over‐the‐line method to prevent perforation [10]. The present case differs from previous reports in two respects: the use of a CAST hood with a slim upper gastrointestinal endoscope and the method of hemostasis. In both previous reports, colonoscopy was initiated with the CAST hood already attached. However, attachment of the CAST hood may narrow the endoscopic field of view and reduce visibility during exploration. Furthermore, insertion through a colonic lumen containing a large amount of blood or residual material is not necessarily straightforward, which represents a potential disadvantage of the CAST hood. In the present case, the culprit diverticulum had already been identified using a conventional distal attachment hood and marked with a clip in preparation for EBL. Although blood remained in the colonic lumen when the endoscope was reinserted with the CAST hood attached, the culprit diverticulum was easily reached, partly because the conventional colonoscope had been replaced with a slim endoscope that provided improved handling and maneuverability. Furthermore, the reopenable clip enabled precise targeted hemostasis while maintaining continuous visualization of the culprit vessel. Although prior identification and marking of the culprit diverticulum are prerequisites, this technique requires only a few clips, allows rapid hemostasis, and avoids expensive devices such as OTSC. We also experienced another case in which this technique was effective for rebleeding after indirect clipping (Video S1), suggesting its potential as a practical and cost‐effective salvage hemostasis option.

This report has some limitations. First, this technique requires a certain level of technical expertise. Second, it represents a single case, and patient‐specific anatomical factors and disease conditions may have contributed to the procedural success. Therefore, further accumulation of cases through multicenter studies is needed to validate this technique.

In conclusion, we encountered a case of CDB in which direct clipping with a tapered‐tip hood and a slim endoscope achieved hemostasis after unsuccessful EBL. This approach enabled direct visualization and subsequent direct clipping of an exposed vessel within the diverticulum. This technique may represent a practical and cost‐effective salvage option for hemostasis after unsuccessful EBL.

## Author Contributions

Hirotake Kusumoto conceived the study, managed the case, collected and analyzed the clinical data, and drafted the manuscript. All co‐authors provided clinical support and supervision, and reviewed and approved the final manuscript.

## Funding

The authors have nothing to report.

## Ethics Statement

**Approval of the research protocol by an Institutional Review Board**: N/A.

## Consent

Informed consent for the treatment and publication was obtained from both patients.

## Conflicts of Interest

The authors declare no conflicts of interest.

## Supporting information




**VIDEO S1** Representative video from a different patient demonstrating direct clipping using a tapered‐tip hood and a slim endoscope for colonic diverticular bleeding. After removal of the clips previously placed for indirect clipping, the culprit diverticulum was approached using a tapered‐tip hood and a slim endoscope. Continuous water irrigation enabled direct visualization of the exposed vessel within the diverticulum, followed by successful direct clipping.

## Data Availability

The data that support the findings of this study are available from the corresponding author upon reasonable request.
